# Chlorido{4-cyclo­hexyl-1-[1-(pyridin-2-yl-κ*N*)ethyl­idene]thio­semicarbazidato-κ^2^ *N* ^1^,*S*}diphenyl­tin(IV)

**DOI:** 10.1107/S1600536812010902

**Published:** 2012-03-17

**Authors:** Md. Abu Affan, Md. Abdus Salam, Ismail Jusoh, Seik Weng Ng, Edward R. T. Tiekink

**Affiliations:** a Faculty of Resource Science and Technology, Universiti Malaysia Sarawak, 94300 Kota Samaharan, Sawarak, Malaysia; b Department of Chemistry, University of Malaya, 50603 Kuala Lumpur, Malaysia; c Chemistry Department, Faculty of Science, King Abdulaziz University, PO Box 80203 Jeddah, Saudi Arabia

## Abstract

The distorted octa­hedral geometry about the Sn^IV^ atom in the title compound, [Sn(C_6_H_5_)_2_(C_14_H_19_N_4_S)Cl], is defined by the *N*,*N*,*S*-tridentate Schiff base ligand, two mutually *trans ipso*-C atoms of the Sn-bound phenyl groups, and the Cl atom which is *trans* to the azo N atom. The two five-membered chelate rings and pyridyl ring are almost coplanar with the dihedral angle between the outer five-membered chelate and pyridine rings being 5.39 (8)°. Centrosymmetric dimers feature in the crystal packing mediated by N—H⋯S hydrogen bonds, leading to eight-membered {⋯HNCS}_2_ synthons. The dimeric aggregates are connected into a three-dimensional architecture by C—H⋯Cl and C—H⋯π inter­actions, as well as π–π inter­actions occurring between centrosymmetrically related pyridine rings [centroid–centroid distance = 3.6322 (13) Å].

## Related literature

For the crystal structure of the dichloridophenyl analogue, see: Salam *et al.* (2010 ▶). For a related structure, see: de Sousa *et al.* (2007 ▶).
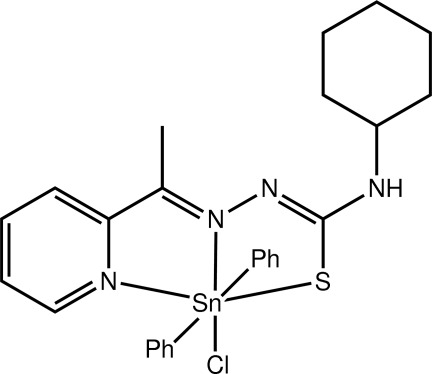


## Experimental

### 

#### Crystal data

[Sn(C_6_H_5_)_2_(C_14_H_19_N_4_S)Cl]
*M* *_r_* = 583.73
Triclinic, 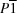

*a* = 9.7368 (4) Å
*b* = 9.9771 (4) Å
*c* = 13.4045 (5) Å
α = 90.103 (3)°
β = 97.013 (3)°
γ = 100.931 (4)°
*V* = 1268.57 (9) Å^3^
*Z* = 2
Mo *K*α radiation
μ = 1.22 mm^−1^
*T* = 100 K
0.40 × 0.30 × 0.20 mm

#### Data collection

Agilent SuperNova Dual diffractometer with an Atlas detector
Absorption correction: multi-scan (*CrysAlis PRO*; Agilent, 2011 ▶) *T*_min_ = 0.642, *T*_max_ = 0.793
8973 measured reflections
5781 independent reflections
5122 reflections with *I* > 2σ(*I*)
*R*_int_ = 0.029

#### Refinement

*R*[*F* ^2^ > 2σ(*F* ^2^)] = 0.029
*wR*(*F* ^2^) = 0.061
*S* = 1.00
5781 reflections
299 parameters
H-atom parameters constrained
Δρ_max_ = 0.51 e Å^−3^
Δρ_min_ = −0.58 e Å^−3^

### 

Data collection: *CrysAlis PRO* (Agilent, 2011 ▶); cell refinement: *CrysAlis PRO*; data reduction: *CrysAlis PRO*; program(s) used to solve structure: *SHELXS97* (Sheldrick, 2008 ▶); program(s) used to refine structure: *SHELXL97* (Sheldrick, 2008 ▶); molecular graphics: *X-SEED* (Barbour, 2001 ▶) and *DIAMOND* (Brandenburg, 2006 ▶); software used to prepare material for publication: *publCIF* (Westrip, 2010 ▶).

## Supplementary Material

Crystal structure: contains datablock(s) global, I. DOI: 10.1107/S1600536812010902/zl2462sup1.cif

Structure factors: contains datablock(s) I. DOI: 10.1107/S1600536812010902/zl2462Isup2.hkl

Additional supplementary materials:  crystallographic information; 3D view; checkCIF report

## Figures and Tables

**Table 1:** Selected bond lengths (Å)

Sn—C1	2.152 (2)
Sn—C7	2.159 (2)
Sn—N2	2.3100 (19)
Sn—N1	2.3869 (19)
Sn—S1	2.5209 (6)
Sn—Cl1	2.5449 (6)

**Table 2:** Hydrogen-bond geometry (Å, °) *Cg*1 is the centroid of the C7–C12 ring.

*D*—H⋯*A*	*D*—H	H⋯*A*	*D*⋯*A*	*D*—H⋯*A*
N4—H1⋯S1^i^	0.88	2.62	3.489 (2)	171
C13—H13⋯Cl1^ii^	0.95	2.73	3.415 (3)	129
C19—H19*C*⋯Cl1^iii^	0.98	2.85	3.809 (2)	166
C15—H15⋯*Cg*1^iv^	0.95	2.47	3.384 (3)	162

Symmetry codes: (i) 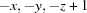; (ii) 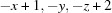; (iii) 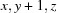; (iv) 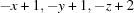.

**Fig. 1.**
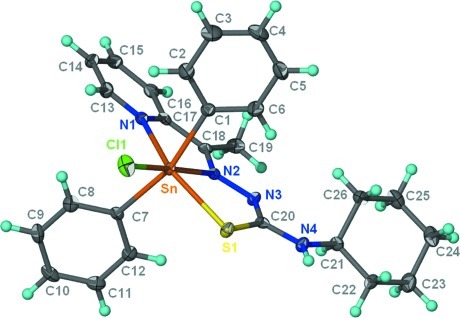
The molecular structure of the title compound showing the atom-labelling scheme and displacement ellipsoids at the 70% probability level.

**Fig. 2.**
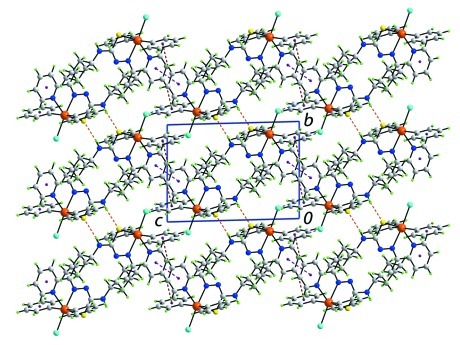
A view in projection down the a axis of the unit-cell contents of the title compound. The N—H···S, C—H···Cl, C—H···π and π—π interactions are shown as orange, blue, brown and purple dashed lines, respectively.

## supplementary crystallographic information

### Comment

The synthesis and crystal structure of the title compound was determined in connection with recent structural studies of organotin chlorido derivatives of thiosemicarbazones (Salam *et al.*, 2010).

The Sn atom in the title compound, Fig. 1, exists within a six atom C_2_ClN_2_S donor set defined by the tridentate monodeprotonated Schiff base ligand, two mutually *trans ipso*-C atoms of the Sn-bound phenyl groups, and the Cl atom which is *trans* to the azo-N atom, Table 1. There are distortions from the ideal octahedral geometry which are ascribed to the restricted bite angles formed by the Schiff base ligand which result in an angle of 145.90 (5)° for the nominally *trans* S1—–Sn—–N1 angle. The disposition of donor atoms resembles that found in the structure of the *N*-4-morpholinyl derivative (de Sousa *et al.*, 2007). Both five-membered rings are essentially planar with the r.m.s. deviations being 0.111 and 0.020 Å for the SnSN_2_C and SnN_2_C_2_ rings, respectively; the former ring has a small twist about the Sn—S1 bond with Sn and S1 atoms lying 0.068 (1) and -0.081 (1) Å out of the least-squares plane, respectively. The dihedral angle between the chelate rings is 3.42 (7)° and those between each of these and the pyridyl ring are 5.39 (8) and 2.29 (9)°, respectively, indicating an essentially planar arrangement of fused rings. Finally, the Sn-bound benzene rings are almost parallel with the dihedral angle being 8.72 (12)°.

The most significant feature in the crystal packing of the title compound is the formation of centrosymmetric dimers *via* N—H···S hydrogen bonds that lead to flat, eight-membered {···HNCS}_2_ synthons, Table 1. The dimeric aggregates are connected into a three dimensional architecture by C—H···Cl and C—H···π interactions, Table 1, as well as π—π interactions occurring between centrosymmetrically related pyridyl rings [centroid···centroid distance = 3.6322 (13) Å for symmetry operation: 1 - *x*, 1 - *y*, 2 - *z*], Fig. 2.

### Experimental

2-Acetylpyridine-*N*-cyclohexylthiosemicarbazone (0.28 g, 1 mmol) was dissolved in methanol (10 ml) in a Schlenk flask under a nitrogen atmosphere. Diphenyltin(IV) dichloride (0.34 g, 1 mmol) dissolved in methanol (10 ml) was added. The yellow solution was refluxed for 4 h. Slow evaporation of the solvent gave a yellow compound (0.423 g). Recrystallization from a chloroform/methanol (1/1) mixture gave small dark-yellow prisms embedded in large light-yellow blocks. A small light-yellow specimen was cut from a light-yellow block for the diffraction measurements. The dark-yellow specimen proved to be (C_6_H_5_)Sn(C_14_H_19_N_4_S)Cl_2_ from unit cell determination (Salam *et al.*, 2010).

### Refinement

Carbon-bound H-atoms were placed in calculated positions [C—H = 0.95 to 1.00 Å, *U*_iso_(H) = 1.2 to 1.5*U*_eq_(C)] and were included in the refinement in the riding model approximation. The amino H-atom was similarly treated [N—H = 0.88 Å with *U*_iso_(H) = 1.2*U*_eq_(N)]. Owing to poor agreement, several reflections, *i.e*. (2 6 8), (2 5 8), (2 6 7) and (2 4 8), were omitted from the final refinement.

### Crystal data

**Table:**

[Sn(C_6_H_5_)_2_(C_14_H_19_N_4_S)Cl]	*Z* = 2
*M**_r_* = 583.73	*F*(000) = 592
Triclinic, *P*1	*D*_x_ = 1.528 Mg m^−^^3^
Hall symbol: -P 1	Mo *K*α radiation, λ = 0.71073 Å
*a* = 9.7368 (4) Å	Cell parameters from 5479 reflections
*b* = 9.9771 (4) Å	θ = 2.5–27.5°
*c* = 13.4045 (5) Å	µ = 1.22 mm^−^^1^
α = 90.103 (3)°	*T* = 100 K
β = 97.013 (3)°	Irregular, light-yellow
γ = 100.931 (4)°	0.40 × 0.30 × 0.20 mm
*V* = 1268.57 (9) Å^3^	

### Data collection

**Table:**

Agilent SuperNova Dual diffractometer with an Atlas detector	5781 independent reflections
Radiation source: SuperNova (Mo) X-ray Source	5122 reflections with *I* > 2σ(*I*)
Mirror monochromator	*R*_int_ = 0.029
Detector resolution: 10.4041 pixels mm^-1^	θ_max_ = 27.6°, θ_min_ = 2.5°
ω scan	*h* = −12→12
Absorption correction: multi-scan (*CrysAlis PRO*; Agilent, 2011)	*k* = −10→12
*T*_min_ = 0.642, *T*_max_ = 0.793	*l* = −17→17
8973 measured reflections	

### Refinement

**Table:**

Refinement on *F*^2^	Primary atom site location: structure-invariant direct methods
Least-squares matrix: full	Secondary atom site location: difference Fourier map
*R*[*F*^2^ > 2σ(*F*^2^)] = 0.029	Hydrogen site location: inferred from neighbouring sites
*wR*(*F*^2^) = 0.061	H-atom parameters constrained
*S* = 1.00	*w* = 1/[σ^2^(*F*_o_^2^) + (0.0216*P*)^2^] where *P* = (*F*_o_^2^ + 2*F*_c_^2^)/3
5781 reflections	(Δ/σ)_max_ = 0.001
299 parameters	Δρ_max_ = 0.51 e Å^−^^3^
0 restraints	Δρ_min_ = −0.58 e Å^−^^3^

### Fractional atomic coordinates and isotropic or equivalent isotropic displacement parameters (Å^2^)

**Table:**

	*x*	*y*	*z*	*U*_iso_*/*U*_eq_	
Sn	0.336547 (16)	0.124100 (16)	0.775567 (11)	0.01076 (5)	
Cl1	0.34918 (7)	−0.11363 (6)	0.83991 (5)	0.02221 (14)	
S1	0.13930 (6)	0.06101 (6)	0.63351 (4)	0.01420 (13)	
N1	0.49818 (19)	0.3058 (2)	0.86673 (14)	0.0128 (4)	
N2	0.31702 (19)	0.33125 (19)	0.70413 (14)	0.0111 (4)	
N3	0.23447 (19)	0.3390 (2)	0.61378 (14)	0.0130 (4)	
N4	0.08466 (19)	0.2234 (2)	0.48652 (14)	0.0144 (4)	
H1	0.0336	0.1458	0.4612	0.017*	
C1	0.5121 (2)	0.1152 (2)	0.69424 (17)	0.0139 (5)	
C2	0.6324 (3)	0.0724 (3)	0.74073 (19)	0.0227 (6)	
H2	0.6366	0.0452	0.8087	0.027*	
C3	0.7461 (3)	0.0697 (3)	0.6874 (2)	0.0272 (6)	
H3	0.8280	0.0414	0.7195	0.033*	
C4	0.7409 (3)	0.1074 (3)	0.58891 (19)	0.0226 (6)	
H4	0.8189	0.1052	0.5531	0.027*	
C5	0.6220 (3)	0.1485 (3)	0.54170 (19)	0.0222 (6)	
H5	0.6178	0.1733	0.4732	0.027*	
C6	0.5085 (3)	0.1535 (3)	0.59448 (18)	0.0190 (5)	
H6	0.4278	0.1835	0.5620	0.023*	
C7	0.2074 (2)	0.1485 (2)	0.89180 (17)	0.0118 (5)	
C8	0.2437 (3)	0.1153 (2)	0.99069 (17)	0.0173 (5)	
H8	0.3282	0.0817	1.0086	0.021*	
C9	0.1577 (3)	0.1305 (2)	1.06400 (18)	0.0187 (5)	
H9	0.1843	0.1085	1.1316	0.022*	
C10	0.0328 (3)	0.1780 (2)	1.03850 (19)	0.0189 (5)	
H10	−0.0268	0.1868	1.0882	0.023*	
C11	−0.0035 (2)	0.2120 (2)	0.94064 (18)	0.0184 (5)	
H11	−0.0885	0.2447	0.9230	0.022*	
C12	0.0832 (2)	0.1989 (2)	0.86723 (18)	0.0152 (5)	
H12	0.0579	0.2243	0.8002	0.018*	
C13	0.5906 (2)	0.2890 (3)	0.94570 (17)	0.0162 (5)	
H13	0.5921	0.1992	0.9687	0.019*	
C14	0.6845 (2)	0.3973 (3)	0.99555 (18)	0.0171 (5)	
H14	0.7496	0.3821	1.0512	0.021*	
C15	0.6809 (2)	0.5272 (3)	0.96253 (18)	0.0176 (5)	
H15	0.7435	0.6035	0.9955	0.021*	
C16	0.5851 (2)	0.5460 (2)	0.88041 (17)	0.0150 (5)	
H16	0.5808	0.6352	0.8572	0.018*	
C17	0.4954 (2)	0.4324 (2)	0.83251 (17)	0.0128 (5)	
C18	0.3979 (2)	0.4439 (2)	0.74072 (17)	0.0128 (5)	
C19	0.4031 (2)	0.5785 (2)	0.69161 (18)	0.0173 (5)	
H19A	0.3328	0.5687	0.6319	0.026*	
H19B	0.4973	0.6103	0.6718	0.026*	
H19C	0.3828	0.6450	0.7389	0.026*	
C20	0.1583 (2)	0.2215 (2)	0.57772 (17)	0.0125 (5)	
C21	0.0833 (2)	0.3452 (2)	0.42632 (17)	0.0139 (5)	
H21	0.0663	0.4201	0.4702	0.017*	
C22	−0.0388 (2)	0.3141 (3)	0.34146 (18)	0.0179 (5)	
H22A	−0.0269	0.2359	0.3000	0.022*	
H22B	−0.1285	0.2879	0.3705	0.022*	
C23	−0.0460 (3)	0.4371 (3)	0.27500 (19)	0.0235 (6)	
H23A	−0.1208	0.4111	0.2176	0.028*	
H23B	−0.0713	0.5110	0.3142	0.028*	
C24	0.0936 (3)	0.4897 (3)	0.23527 (18)	0.0224 (6)	
H24A	0.0875	0.5736	0.1971	0.027*	
H24B	0.1133	0.4203	0.1890	0.027*	
C25	0.2134 (3)	0.5208 (3)	0.32162 (19)	0.0222 (6)	
H25A	0.3037	0.5521	0.2943	0.027*	
H25B	0.1972	0.5950	0.3652	0.027*	
C26	0.2223 (2)	0.3940 (3)	0.38360 (18)	0.0175 (5)	
H26A	0.3003	0.4156	0.4393	0.021*	
H26B	0.2419	0.3207	0.3407	0.021*	

### Atomic displacement parameters (Å^2^)

**Table:**

	*U*^11^	*U*^22^	*U*^33^	*U*^12^	*U*^13^	*U*^23^
Sn	0.01145 (9)	0.01048 (9)	0.01016 (9)	0.00200 (6)	0.00073 (6)	−0.00036 (6)
Cl1	0.0329 (4)	0.0148 (3)	0.0222 (3)	0.0097 (3)	0.0083 (3)	0.0050 (2)
S1	0.0159 (3)	0.0116 (3)	0.0131 (3)	0.0000 (2)	−0.0020 (2)	0.0002 (2)
N1	0.0120 (10)	0.0146 (10)	0.0119 (10)	0.0019 (8)	0.0025 (8)	−0.0009 (8)
N2	0.0097 (9)	0.0130 (10)	0.0109 (9)	0.0024 (8)	0.0019 (8)	0.0010 (8)
N3	0.0124 (10)	0.0128 (10)	0.0126 (10)	0.0018 (8)	−0.0021 (8)	0.0013 (8)
N4	0.0152 (10)	0.0128 (10)	0.0127 (10)	−0.0013 (8)	−0.0017 (8)	0.0013 (8)
C1	0.0124 (12)	0.0134 (12)	0.0148 (12)	−0.0001 (10)	0.0016 (9)	−0.0014 (10)
C2	0.0195 (13)	0.0308 (16)	0.0201 (14)	0.0093 (12)	0.0041 (11)	0.0063 (12)
C3	0.0187 (14)	0.0354 (17)	0.0311 (16)	0.0123 (13)	0.0054 (12)	0.0020 (13)
C4	0.0183 (13)	0.0228 (14)	0.0276 (15)	0.0014 (11)	0.0108 (11)	−0.0054 (11)
C5	0.0243 (14)	0.0253 (15)	0.0168 (13)	0.0014 (12)	0.0074 (11)	0.0002 (11)
C6	0.0166 (13)	0.0228 (14)	0.0188 (13)	0.0072 (11)	0.0017 (10)	0.0000 (11)
C7	0.0122 (11)	0.0097 (11)	0.0124 (11)	−0.0019 (9)	0.0030 (9)	−0.0020 (9)
C8	0.0194 (13)	0.0150 (13)	0.0175 (13)	0.0035 (10)	0.0022 (10)	0.0001 (10)
C9	0.0258 (14)	0.0164 (13)	0.0119 (12)	−0.0011 (11)	0.0030 (10)	0.0015 (10)
C10	0.0197 (13)	0.0147 (13)	0.0229 (13)	−0.0004 (11)	0.0111 (11)	−0.0037 (10)
C11	0.0139 (12)	0.0172 (13)	0.0247 (14)	0.0034 (10)	0.0037 (10)	−0.0019 (11)
C12	0.0158 (12)	0.0129 (12)	0.0161 (12)	0.0014 (10)	0.0004 (10)	0.0010 (10)
C13	0.0173 (12)	0.0201 (13)	0.0117 (12)	0.0050 (11)	0.0014 (10)	−0.0015 (10)
C14	0.0133 (12)	0.0250 (14)	0.0119 (12)	0.0019 (11)	−0.0007 (9)	−0.0028 (10)
C15	0.0141 (12)	0.0201 (13)	0.0163 (12)	−0.0030 (10)	0.0030 (10)	−0.0042 (10)
C16	0.0144 (12)	0.0138 (12)	0.0164 (12)	0.0001 (10)	0.0042 (10)	−0.0007 (10)
C17	0.0119 (11)	0.0145 (12)	0.0128 (11)	0.0018 (10)	0.0060 (9)	0.0004 (9)
C18	0.0088 (11)	0.0153 (12)	0.0149 (12)	0.0025 (10)	0.0034 (9)	−0.0014 (10)
C19	0.0155 (12)	0.0123 (12)	0.0236 (13)	0.0009 (10)	0.0033 (10)	0.0004 (10)
C20	0.0095 (11)	0.0153 (12)	0.0140 (12)	0.0040 (10)	0.0034 (9)	−0.0001 (9)
C21	0.0139 (12)	0.0124 (12)	0.0151 (12)	0.0013 (10)	0.0026 (9)	0.0043 (9)
C22	0.0151 (12)	0.0233 (14)	0.0147 (12)	0.0039 (11)	−0.0015 (10)	0.0046 (10)
C23	0.0264 (14)	0.0284 (15)	0.0183 (13)	0.0127 (12)	0.0018 (11)	0.0063 (11)
C24	0.0300 (15)	0.0214 (14)	0.0190 (13)	0.0096 (12)	0.0083 (11)	0.0070 (11)
C25	0.0263 (14)	0.0204 (14)	0.0212 (14)	0.0027 (12)	0.0101 (11)	0.0016 (11)
C26	0.0142 (12)	0.0208 (13)	0.0172 (12)	0.0019 (10)	0.0035 (10)	0.0011 (10)

### Geometric parameters (Å, º)

**Table:**

Sn—C1	2.152 (2)	C11—C12	1.394 (3)
Sn—C7	2.159 (2)	C11—H11	0.9500
Sn—N2	2.3100 (19)	C12—H12	0.9500
Sn—N1	2.3869 (19)	C13—C14	1.387 (3)
Sn—S1	2.5209 (6)	C13—H13	0.9500
Sn—Cl1	2.5449 (6)	C14—C15	1.376 (3)
S1—C20	1.756 (2)	C14—H14	0.9500
N1—C13	1.335 (3)	C15—C16	1.391 (3)
N1—C17	1.349 (3)	C15—H15	0.9500
N2—C18	1.300 (3)	C16—C17	1.394 (3)
N2—N3	1.380 (3)	C16—H16	0.9500
N3—C20	1.319 (3)	C17—C18	1.478 (3)
N4—C20	1.342 (3)	C18—C19	1.492 (3)
N4—C21	1.461 (3)	C19—H19A	0.9800
N4—H1	0.8800	C19—H19B	0.9800
C1—C6	1.390 (3)	C19—H19C	0.9800
C1—C2	1.398 (3)	C21—C22	1.527 (3)
C2—C3	1.394 (3)	C21—C26	1.529 (3)
C2—H2	0.9500	C21—H21	1.0000
C3—C4	1.370 (4)	C22—C23	1.525 (3)
C3—H3	0.9500	C22—H22A	0.9900
C4—C5	1.382 (3)	C22—H22B	0.9900
C4—H4	0.9500	C23—C24	1.521 (3)
C5—C6	1.391 (3)	C23—H23A	0.9900
C5—H5	0.9500	C23—H23B	0.9900
C6—H6	0.9500	C24—C25	1.526 (4)
C7—C8	1.387 (3)	C24—H24A	0.9900
C7—C12	1.399 (3)	C24—H24B	0.9900
C8—C9	1.393 (3)	C25—C26	1.525 (3)
C8—H8	0.9500	C25—H25A	0.9900
C9—C10	1.391 (3)	C25—H25B	0.9900
C9—H9	0.9500	C26—H26A	0.9900
C10—C11	1.377 (3)	C26—H26B	0.9900
C10—H10	0.9500		
			
C1—Sn—C7	163.82 (9)	N1—C13—H13	118.7
C1—Sn—N2	89.52 (8)	C14—C13—H13	118.7
C7—Sn—N2	94.19 (7)	C15—C14—C13	118.4 (2)
C1—Sn—N1	83.36 (7)	C15—C14—H14	120.8
C7—Sn—N1	83.23 (7)	C13—C14—H14	120.8
N2—Sn—N1	69.43 (6)	C14—C15—C16	119.4 (2)
C1—Sn—S1	98.90 (6)	C14—C15—H15	120.3
C7—Sn—S1	97.28 (6)	C16—C15—H15	120.3
N2—Sn—S1	76.55 (5)	C15—C16—C17	119.3 (2)
N1—Sn—S1	145.90 (5)	C15—C16—H16	120.4
C1—Sn—Cl1	89.13 (6)	C17—C16—H16	120.4
C7—Sn—Cl1	88.37 (6)	N1—C17—C16	120.7 (2)
N2—Sn—Cl1	175.15 (5)	N1—C17—C18	117.1 (2)
N1—Sn—Cl1	115.02 (5)	C16—C17—C18	122.1 (2)
S1—Sn—Cl1	99.07 (2)	N2—C18—C17	116.6 (2)
C20—S1—Sn	96.99 (8)	N2—C18—C19	123.7 (2)
C13—N1—C17	119.5 (2)	C17—C18—C19	119.6 (2)
C13—N1—Sn	124.50 (16)	C18—C19—H19A	109.5
C17—N1—Sn	115.98 (15)	C18—C19—H19B	109.5
C18—N2—N3	116.92 (19)	H19A—C19—H19B	109.5
C18—N2—Sn	120.81 (15)	C18—C19—H19C	109.5
N3—N2—Sn	121.65 (14)	H19A—C19—H19C	109.5
C20—N3—N2	114.79 (19)	H19B—C19—H19C	109.5
C20—N4—C21	124.7 (2)	N3—C20—N4	116.4 (2)
C20—N4—H1	117.7	N3—C20—S1	128.57 (18)
C21—N4—H1	117.7	N4—C20—S1	114.99 (17)
C6—C1—C2	118.7 (2)	N4—C21—C22	108.65 (19)
C6—C1—Sn	120.44 (17)	N4—C21—C26	112.62 (19)
C2—C1—Sn	120.87 (17)	C22—C21—C26	110.39 (19)
C3—C2—C1	120.1 (2)	N4—C21—H21	108.4
C3—C2—H2	120.0	C22—C21—H21	108.4
C1—C2—H2	120.0	C26—C21—H21	108.4
C4—C3—C2	120.6 (2)	C23—C22—C21	111.4 (2)
C4—C3—H3	119.7	C23—C22—H22A	109.3
C2—C3—H3	119.7	C21—C22—H22A	109.3
C3—C4—C5	120.0 (2)	C23—C22—H22B	109.3
C3—C4—H4	120.0	C21—C22—H22B	109.3
C5—C4—H4	120.0	H22A—C22—H22B	108.0
C4—C5—C6	120.0 (2)	C24—C23—C22	111.7 (2)
C4—C5—H5	120.0	C24—C23—H23A	109.3
C6—C5—H5	120.0	C22—C23—H23A	109.3
C5—C6—C1	120.7 (2)	C24—C23—H23B	109.3
C5—C6—H6	119.7	C22—C23—H23B	109.3
C1—C6—H6	119.7	H23A—C23—H23B	107.9
C8—C7—C12	118.7 (2)	C23—C24—C25	110.7 (2)
C8—C7—Sn	121.86 (17)	C23—C24—H24A	109.5
C12—C7—Sn	119.42 (17)	C25—C24—H24A	109.5
C7—C8—C9	120.7 (2)	C23—C24—H24B	109.5
C7—C8—H8	119.6	C25—C24—H24B	109.5
C9—C8—H8	119.6	H24A—C24—H24B	108.1
C10—C9—C8	120.2 (2)	C26—C25—C24	110.5 (2)
C10—C9—H9	119.9	C26—C25—H25A	109.5
C8—C9—H9	119.9	C24—C25—H25A	109.5
C11—C10—C9	119.4 (2)	C26—C25—H25B	109.5
C11—C10—H10	120.3	C24—C25—H25B	109.5
C9—C10—H10	120.3	H25A—C25—H25B	108.1
C10—C11—C12	120.6 (2)	C25—C26—C21	109.84 (19)
C10—C11—H11	119.7	C25—C26—H26A	109.7
C12—C11—H11	119.7	C21—C26—H26A	109.7
C11—C12—C7	120.3 (2)	C25—C26—H26B	109.7
C11—C12—H12	119.9	C21—C26—H26B	109.7
C7—C12—H12	119.9	H26A—C26—H26B	108.2
N1—C13—C14	122.7 (2)		
			
C1—Sn—S1—C20	−78.66 (10)	C1—Sn—C7—C12	146.1 (3)
C7—Sn—S1—C20	101.29 (9)	N2—Sn—C7—C12	43.22 (19)
N2—Sn—S1—C20	8.70 (8)	N1—Sn—C7—C12	111.91 (18)
N1—Sn—S1—C20	12.62 (11)	S1—Sn—C7—C12	−33.73 (18)
Cl1—Sn—S1—C20	−169.21 (7)	Cl1—Sn—C7—C12	−132.66 (18)
C1—Sn—N1—C13	−85.65 (17)	C12—C7—C8—C9	0.6 (4)
C7—Sn—N1—C13	85.28 (17)	Sn—C7—C8—C9	−179.28 (17)
N2—Sn—N1—C13	−177.64 (18)	C7—C8—C9—C10	0.8 (4)
S1—Sn—N1—C13	178.28 (13)	C8—C9—C10—C11	−1.2 (4)
Cl1—Sn—N1—C13	0.27 (18)	C9—C10—C11—C12	0.2 (4)
C1—Sn—N1—C17	92.66 (16)	C10—C11—C12—C7	1.2 (4)
C7—Sn—N1—C17	−96.41 (16)	C8—C7—C12—C11	−1.5 (3)
N2—Sn—N1—C17	0.67 (14)	Sn—C7—C12—C11	178.33 (17)
S1—Sn—N1—C17	−3.4 (2)	C17—N1—C13—C14	0.6 (3)
Cl1—Sn—N1—C17	178.58 (13)	Sn—N1—C13—C14	178.85 (16)
C1—Sn—N2—C18	−81.56 (17)	N1—C13—C14—C15	0.6 (3)
C7—Sn—N2—C18	82.68 (17)	C13—C14—C15—C16	−0.5 (3)
N1—Sn—N2—C18	1.53 (15)	C14—C15—C16—C17	−0.7 (3)
S1—Sn—N2—C18	179.18 (17)	C13—N1—C17—C16	−1.8 (3)
C1—Sn—N2—N3	89.24 (15)	Sn—N1—C17—C16	179.77 (15)
C7—Sn—N2—N3	−106.53 (15)	C13—N1—C17—C18	175.89 (18)
N1—Sn—N2—N3	172.32 (16)	Sn—N1—C17—C18	−2.5 (2)
S1—Sn—N2—N3	−10.03 (13)	C15—C16—C17—N1	1.9 (3)
C18—N2—N3—C20	177.56 (19)	C15—C16—C17—C18	−175.72 (19)
Sn—N2—N3—C20	6.4 (2)	N3—N2—C18—C17	−174.55 (17)
C7—Sn—C1—C6	−145.4 (3)	Sn—N2—C18—C17	−3.3 (3)
N2—Sn—C1—C6	−41.8 (2)	N3—N2—C18—C19	1.3 (3)
N1—Sn—C1—C6	−111.2 (2)	Sn—N2—C18—C19	172.46 (16)
S1—Sn—C1—C6	34.5 (2)	N1—C17—C18—N2	3.9 (3)
Cl1—Sn—C1—C6	133.5 (2)	C16—C17—C18—N2	−178.5 (2)
C7—Sn—C1—C2	33.9 (4)	N1—C17—C18—C19	−172.12 (19)
N2—Sn—C1—C2	137.4 (2)	C16—C17—C18—C19	5.6 (3)
N1—Sn—C1—C2	68.1 (2)	N2—N3—C20—N4	−175.27 (17)
S1—Sn—C1—C2	−146.28 (19)	N2—N3—C20—S1	4.9 (3)
Cl1—Sn—C1—C2	−47.2 (2)	C21—N4—C20—N3	−0.3 (3)
C6—C1—C2—C3	0.4 (4)	C21—N4—C20—S1	179.50 (16)
Sn—C1—C2—C3	−178.8 (2)	Sn—S1—C20—N3	−11.4 (2)
C1—C2—C3—C4	−0.6 (4)	Sn—S1—C20—N4	168.86 (15)
C2—C3—C4—C5	0.0 (4)	C20—N4—C21—C22	−165.9 (2)
C3—C4—C5—C6	0.9 (4)	C20—N4—C21—C26	71.5 (3)
C4—C5—C6—C1	−1.2 (4)	N4—C21—C22—C23	−179.67 (18)
C2—C1—C6—C5	0.5 (4)	C26—C21—C22—C23	−55.7 (3)
Sn—C1—C6—C5	179.74 (18)	C21—C22—C23—C24	54.1 (3)
C1—Sn—C7—C8	−34.0 (4)	C22—C23—C24—C25	−54.7 (3)
N2—Sn—C7—C8	−136.91 (19)	C23—C24—C25—C26	57.5 (3)
N1—Sn—C7—C8	−68.22 (19)	C24—C25—C26—C21	−59.4 (3)
S1—Sn—C7—C8	146.14 (19)	N4—C21—C26—C25	179.88 (19)
Cl1—Sn—C7—C8	47.21 (19)	C22—C21—C26—C25	58.2 (3)

### Hydrogen-bond geometry (Å, º)

Cg1 is the centroid of the C7–C12 ring.

**Table:**

*D*—H···*A*	*D*—H	H···*A*	*D*···*A*	*D*—H···*A*
N4—H1···S1^i^	0.88	2.62	3.489 (2)	171
C13—H13···Cl1^ii^	0.95	2.73	3.415 (3)	129
C19—H19*C*···Cl1^iii^	0.98	2.85	3.809 (2)	166
C15—H15···*Cg*1^iv^	0.95	2.47	3.384 (3)	162

Symmetry codes: (i) −*x*, −*y*, −*z*+1; (ii) −*x*+1, −*y*, −*z*+2; (iii) *x*, *y*+1, *z*; (iv) −*x*+1, −*y*+1, −*z*+2.
